# Young Man with Hepatomegaly: A Case of Glycogenic Hepatopathy

**DOI:** 10.1155/2018/6037530

**Published:** 2018-04-15

**Authors:** Walid Abboud, Saif Abdulla, Mohammed Al Zaabi, Ramzi Moufarrej

**Affiliations:** ^1^Department of Internal Medicine, Zayed Military Hospital, Abu Dhabi, UAE; ^2^Department of Gastroenterology, Zayed Military Hospital, Abu Dhabi, UAE; ^3^Department of Critical Care, Zayed Military Hospital, Abu Dhabi, UAE

## Abstract

Glycogenic hepatopathy is a rare but potentially reversible condition characterized by hepatomegaly and elevated liver enzymes occurring in poorly controlled type 1 diabetes mellitus patients and often requires a liver biopsy to confirm the diagnosis. We present the case of a young man who was admitted with diabetic ketoacidosis in the setting of poorly controlled diabetes mellitus type 1 and was noted to have significantly elevated transaminases that continued to worsen despite appropriate treatment of the diabetic ketoacidosis. A liver biopsy confirmed the diagnosis of glycogenic hepatopathy and the patient improved with diabetes control. The aim of this report is to shed light on possible causes of significant elevation of liver enzymes in patients presenting with diabetic ketoacidosis. In addition, we would like to raise awareness about the diagnosis, management, and prognosis of glycogenic hepatopathy and how to differentiate it from other hepatic conditions that have a similar presentation.

## 1. Introduction

Glycogenic hepatopathy presents with abdominal pain, hepatomegaly, and transaminasemia as a result of increased glycogen formation and deposition in the setting of excess levels of insulin and glucose. This condition remains poorly recognized among clinicians, which may lead to expensive and unnecessary testing and delaying appropriate management. In this case report, we reviewed the presentation, evaluation, and management of a patient who developed elevated liver enzymes after initiation of treatment of diabetic ketoacidosis. In addition, we present the thought process involved in the differential diagnosis and workup done for our patient leading to the final diagnosis of glycogenic hepatopathy.

## 2. Case Presentation

A 15-year-old boy was brought to the emergency department by his family for evaluation of abdominal pain associated with nausea and vomiting. His discomfort started one day earlier and was localized to the right upper quadrant of the abdomen. He described a constant pressure unrelated to food intake. He reported no change in urine or stools and no melena, dysphagia, anorexia, increase in abdominal girth, or change in his weight. He was lethargic and fatigued but had no pruritus, jaundice, night sweats, fever, easy bruising, or bleeding.

The patient's medical history was notable for type 1 diabetes mellitus for 6 years, which has been poorly controlled and was complicated by several episodes of diabetic ketoacidosis. There was no family history of diabetes mellitus, autoimmune diseases, or liver disorders. His medications included insulin Lantus 22 units at night and insulin Humalog 20 units three times daily before each meal. He admitted that he had not been consistently compliant with his medications. There was no history of new medication usage. He also denied the use of any over-the-counter medication or alcohol use. There was no recent travel and no sick contact. On physical examination, the patient was noted to be thin and in no acute distress and afebrile with heart rate of 109 beats per minute and blood pressure of 109/60 mm Hg. His weight was 37 kg and his height was 153 cm with a body mass index (BMI) of 15.8 kg/m^2^. The jugular venous pressure was not elevated. Examination of the heart and lungs was unremarkable. Abdominal examination revealed mildly distended abdomen with mild tenderness on palpation in the right upper quadrant. There was no rebound or guarding and Murphy's sign was absent. The liver was palpable 6 cm below the costal margin, with a smooth edge. The spleen was not palpable, and there was no evidence of ascites. Skin examination showed no palmar erythema, leg edema, spider angiomata, or evidence of pigmentation. There was a scant presence of axillary and pubic hair. The neurologic examination, including mental status, was normal.

## 3. Investigations

On presentation, blood glucose was 480 mg per deciliter (mg/dl). The serum sodium level was 137 mmol per liter (mmol/L), potassium was 4.1 mmol/L, chloride was 97 mmol/L, and bicarbonate was 19 mmol/L. The anion gap was calculated at 21. The blood urea nitrogen (BUN) was 29 mg/dl and the creatinine was 0.6 mg/dl. The arterial blood gas showed a pH of 7.25, partial pressure of carbon dioxide (PCO_2_) of 30 mmHg, and partial pressure of oxygen (PO_2_) of 95 mmHg. The liver enzymes were abnormal with the alanine aminotransferase (ALT) of 262 units per liter (U/L) (normal range: 16 to 63 U/L) and aspartate aminotransferase (AST) of 205 U/L (normal range: 0 to 37 U/L). The alkaline phosphatase was 139 U/L (normal range: <322 U/L), gamma-glutamyl transferase (GGT) was 122 U/L (normal range: 15–85 U/L), and the total bilirubin was 0.5 mg/dl (normal range: 0 to 1 mg/dl). Serum albumin was 4.1 g per deciliter and the amylase and lipase levels were within normal ranges. The acetaminophen level was undetectable. His lactate level was 3.2 mmol/L (normal range: 0.4–2 mmol/L). The prothrombin time and partial-thromboplastin time were both normal. The white cell count was 4,700 per cubic millimeter, with a normal differential count. The hemoglobin was 13.8 grams per deciliter, with mean corpuscular volume of 88 fL, and the platelet count was 383,000 per cubic millimeter. The urinalysis showed evidence of ketones. A radiograph of the chest showed no abnormal findings.

## 4. Inpatient Course

The patient was admitted to the hospital with the impression of diabetic ketoacidosis, most probably due to noncompliance with medications. The patient was started on insulin infusion and intravenous fluid with potassium chloride. There was no evidence of sepsis or other causes that precipitated this event. He also had elevated liver enzymes and lactic acid with hepatomegaly, with no obvious etiology.

The patient's ketoacidosis resolved with treatment and he was shifted to his regular dose of insulin with good glycemic control achieved by the second day. He continued to have mild abdominal pain in the right side of the abdomen and his liver enzymes continued to rise significantly. The lactate level also worsened on the second day before slowly trending to normal range on day 7 (Table 1).

Given this rise in liver enzymes associated with abdominal pain and hepatomegaly in the setting of type 1 diabetes mellitus, we initiated the workup to evaluate the etiology behind this presentation. We ordered an ultrasound of the liver to evaluate for evidence of nonalcoholic fatty liver disease. Also, we sent investigation for iron deposition disorder and for autoimmune workup. We also tested for viral hepatitis serology, despite the fact that he had no previous history or risk factors for viral hepatitis. Other conditions considered in our differential diagnosis included medication-induced hepatitis, Wilson's disease, alpha-1 antitrypsin deficiency, glycogen storage disorder, Gaucher's disease, and secondary amyloidosis.

The results of the additional investigations were as follows: the serum iron was 104 *μ*g per deciliter (normal range: 65–176), total iron-binding capacity was 392 *μ*g per deciliter (normal range: 240–450), and ferritin was 141 nanograms per milliliter (normal range: 5–244). His total cholesterol was 218 mg/dl, triglycerides level was 141 mg/dl, high-density lipoprotein (HDL) was 41 mg mg/dl, and low-density lipoprotein (LDL) was 149 mg/dl. The glycated hemoglobin (HbA_1c_) was 12.4%. Hepatitis A, B, and C, Epstein-Barr virus (EBV), and cytomegalovirus (CMV) serology all came back negative. The antimitochondrial antibody and anti-smooth muscle antibody were negative. Results for the ceruloplasmin serum test were in the normal range. The thyroid function was normal. Alpha-1 antitrypsin level was normal. Ultrasonography of the abdomen showed the liver to be enlarged with a bipolar length of 24 cm, with normal echogenicity. The gall bladder had normal wall thickness, with no stones or biliary dilatation. The spleen and kidneys appeared normal.

In conclusion, there was no evidence based on our investigation for the common causes of rising liver enzymes. Revising his previous medical history, the patient had several admissions with diabetic ketoacidosis associated with elevated liver enzymes. Also noted, his liver enzymes continued to rise before ultimately trending down around the ninth hospital day. The decision was then made to perform a liver biopsy.

**Figure 1:**
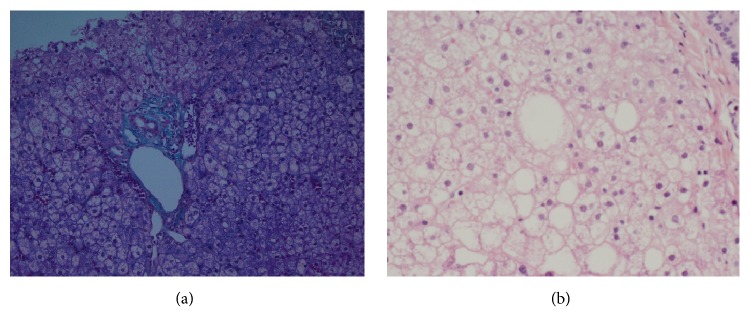
(a) Liver biopsy showing hepatocytic ballooning of moderate degree, with mild microvesicular and macrovesicular steatosis, and prominent glycogenated nuclei by PAS stain (magnification: ×40). (b) Hematoxylin and Eosin staining showing evidence of steatosis, nuclear glycogenosis, and lobular inflammation (magnification: ×100).

The liver biopsy results showed mild macrovesicular and microvesicular steatosis with mild portal and lobular inflammation. Periodic acid-Schiff staining revealed increased overall intracellular glycogen content with the presence of glycogenated nuclei. The biopsy findings were consistent with glycogenic hepatopathy (Figure 1).

## 5. Discussion

Glycogenic hepatopathy was first described in 1930 by Mauriac as “hepatic glycogenosis, characterized by hepatic glycogen deposition in patients with poorly controlled type 1 diabetes mellitus” [1]. Mauriac's original description of this syndrome was for children with features of hepatomegaly, abdominal pain, abnormal liver enzymes, elevated cholesterol, and growth retardation with delayed puberty. However, it was later recognized to occur in both adults [2] and children [3]. In addition, elevation of lactic acid has been increasingly recognized and reported to occur in glycogenic hepatopathy [4].

While the pathophysiology of glycogenic hepatopathy or Mauriac syndrome is not fully understood, the most accepted theory is that the presence of insulin and excess glucose activates glycogen synthase phosphatase into activated glycogen synthase, an enzyme required for the conversion of glucose-1-phosphate to glycogen. This activation promotes glycogen formation and storage in the liver and blocks glycogenolysis, increasing hepatic glycogen stores during hyperglycemia. Insulin is administered as the main treatment for hyperglycemia, driving glycogen synthesis further and inhibiting gluconeogenesis and glycogenolysis, resulting in increasing hepatocyte glycogen stores. Glycogen overload ultimately leads to hepatomegaly and elevated liver enzymes [5, 6]. Elevated levels of glucose and insulin in prepubertal diabetic patients results in a state of hypercortisolism, which explains the pubertal and growth delays [3, 7]. The exact mechanism for lactic acidosis reported in glycogenic hepatopathy is poorly understood [4, 8]. It is felt that the reduction in gluconeogenesis in the liver and the resultant reduction in conversion of pyruvate to glucose may drive the anaerobic reaction of pyruvate to lactate. Therapy with insulin would further reduce gluconeogenesis and increase lactic acid formation, explaining the increase of lactic acidosis observed with early therapy of glycogenic hepatopathy [9]. In a case series conducted by Torbenson et al. which studied 14 patients with biopsy-proven glycogenic hepatopathy, all the patients had type 1 diabetes and 13 patients had abnormal liver-function tests. Hepatomegaly was evident by imaging in 9 patients and 6 patients had elevated HbA_1c_ levels. Steatosis was present in only 2 patients [7].

The diagnosis of glycogenic hepatopathy is usually supported by the following features: poorly controlled type 1 diabetes mellitus, abdominal pain, hepatomegaly, abnormal levels of liver enzymes, and the improvement of the liver enzymes with insulin therapy. The reversibility of the elevation in liver enzymes with rigorous glucose control usually occurs within 2 to 4 weeks [10]. Elevated lactic acid may exist with or without evidence of hypoperfusion [11]. The test to confirm the diagnosis is the liver biopsy. Treatment involves the use of adequate doses of insulin to achieve rigorous glucose control.

It is important to differentiate glycogenic hepatopathy from hepatic steatosis and from other inherited glycogen storage disorders (Tables 2 and 3). While hepatic steatosis is more commonly associated with obesity and type 2 diabetes mellitus and could potentially lead to liver cirrhosis, glycogenic hepatopathy is classically seen in type 1 diabetes mellitus patients and it is highly unlikely to lead to liver cirrhosis [6, 12, 13].

On further follow-up visits, the patient was more compliant with his medical care and his diabetes was under better control with the surveillance of the endocrinology department. He did not show any further rise in his liver enzymes. With HbA_1c_ trending down with treatment, the growth indicators including height, weight, and BMI showed gradual improvement (Table 4).

## 6. Conclusion

Our patient fulfills several criteria for glycogenic hepatopathy, including history of poorly controlled type 1 diabetes mellitus and physical findings of hepatomegaly confirmed by abdominal ultrasonography. In addition, he had elevated liver enzymes and lactic acidosis in the absence of hypoperfusion, both of which responded to insulin therapy and glucose control. With other common etiologies of liver disease such as infectious, autoimmune, metabolic, or drug-induced etiologies ruled out, the characteristic histological findings noted on the liver biopsy showing the increased intracellular glycogen content and the glycogenated nuclei confirmed the diagnosis.

The reversibility of liver enzymes with intensive insulin therapy, in our case, is particularly important given that it is consistent with the diagnosis and prognosis of this condition. This case reinforces the importance of recognizing the features of glycogenic hepatopathy for proper management. Glycogen hepatopathy has an excellent prognosis with improved glycemic control.

## Figures and Tables

**Table 1:** Liver enzymes and lactic acid results.

Tests	Day 1 (admission)	2	3	4	7	8	9	10	*Reference range*
ALT	262	214	226	286	405	433	423	304	*16–63 U/L*
AST	205	345	419	558	678	743	501	310	*0–37 U/L*
Lactate	3.2	5.3	4.7	3.4	1.6				*0.4–2 mmol/L*

**Table 2:** Glycogenic hepatopathy versus hepatic steatosis.

Condition	Glycogenic hepatopathy	Hepatic steatosis
Association	Type 1 diabetes mellitus	Type 2 diabetes mellitus
Treatment	Insulin and better glycemic control	Weight loss
Complication	Unlikely cirrhosis	Complicated by cirrhosis

**Table 3:** Glycogenic hepatopathy versus glycogen storage disorder.

Condition	Glycogenic hepatopathy	Glycogen storage disorder
Etiology	Acquired	Inherited
Course	Reversible	Irreversible

**Table 4:** Height, weight, HbA_1c_, and BMI on follow-up visits.

	7/2015 (admission)	4/2016	2/2017	*Units*
Height cm/(percentile)	153/(8.7)	159/(8.5)	163/(18.7)	*cm*
Weight kg/(percentile)	37/(2.94)	45/(8.85)	54/(22.7)	*kg*
BMI/ (percentile)	15.8/(3.8)	17.8/(18.1)	21.5/(61.8)	*kg/m* ^*2*^
HbA_1c_	12.4	9.8	10.2	*%*
